# Impacts of temperature on giant panda habitat in the north Minshan Mountains

**DOI:** 10.1002/ece3.1901

**Published:** 2016-01-20

**Authors:** Gang Liu, Tianpei Guan, Qiang Dai, Huixin Li, Minghao Gong

**Affiliations:** ^1^ Research Institute of WetlandBeijing Key Laboratory of Wetland Services and Restoration, Chinese Academy of ForestryBeijing100091China; ^2^ Ecoological Security and Protection Key Lab of Sichuan ProvinceMianyang Normal UniversityMianyang621000China; ^3^ Chengdu Institute of BiologyChinese Academy of SciencesChengdu610041China

**Keywords:** Climate change, giant panda, habitat suitability, impacts, temperature

## Abstract

Understanding the impacts of meteorological factors on giant pandas is necessary for future conservation measures in response to global climate change. We integrated temperature data with three main habitat parameters (elevation, vegetation type, and bamboo species) to evaluate the influence of climate change on giant panda habitat in the northern Minshan Mountains using a habitat assessment model. Our study shows that temperature (relative importance = 25.1%) was the second most important variable influencing giant panda habitat excepting the elevation. There was a significant negative correlation between temperature and panda presence (*ρ *= −0.133, *P* < 0.05), and the temperature range preferred by giant pandas within the study area was 18–21°C, followed by 15–17°C and 22–24°C. The overall suitability of giant panda habitats will increase by 2.7%, however, it showed a opposite variation patterns between the eastern and northwestern region of the study area. Suitable and subsuitable habitats in the northwestern region of the study area, which is characterized by higher elevation and latitude, will increase by 18007.8 hm^2^ (9.8% habitat suitability), while the eastern region will suffer a decrease of 9543.5 hm^2^ (7.1% habitat suitability). Our results suggest that increasing areas of suitable giant panda habitat will support future giant panda expansion, and food shortage and insufficient living space will not arise as problems in the northwest Minshan Mountains, which means that giant pandas can adapt to climate change, and therefore may be resilient to climate change. Thus, for the safety and survival of giant pandas in the Baishuijiang Reserve, we propose strengthening the giant panda monitoring program in the west and improving the integrity of habitats to promote population dispersal with adjacent populations in the east.

## Introduction

Climate change is causing changes in species distribution patterns, habitat loss, and even local extinction, and thus threatens ecological safety and global biodiversity (Araújo and Rahbek 2006; Guralnick 2006, Maclean and Wilson 2011). The global temperature has risen approximately 0.74°C over the 20th century, and the current rise continues to increase exponentially (Christensen et al. 2007). Range‐restricted, high‐altitude species are particularly vulnerable to changing climatic conditions because they are commonly characterized by poor dispersal ability, limited food resources, and historically driven habitat fragmentation (Crabtree and Ellis 2010; Chen et al. 2011; Thomas Dirnboock and Rabitsch 2011). In addition to these traits, the giant panda (*Ailuropoda melanoleuca*) feeds almost exclusively on bamboo, has a narrow habitat range, and maintains a low reproductive rate. These features suggest that this species is highly susceptible to climate change (Wang et al. 2010a; Li et al. 2015).

Previous studies have applied various models to project the impacts of climate change on giant pandas, including food shortage, habitat loss, and shifts in population and habitat distributions (Tuanmu et al. 2013; Li et al. 2014). Most of the results of such models have indicated adverse impacts, however, divergent results have caused a lack of valuable conservation guidance. For example, several studies that neglected the influence of biotic and topographic factors have reported overestimations of habitat loss from 37% to 62% (Songer et al. 2012; Fan et al. 2014). Currently, there is no information available on the climatic preferences of giant pandas. Most studies have incorporated many climatic parameters into a single analysis without discrimination, making it impossible to determine the function of a specific variable (Loehle 2011). The more parameters that are incorporated into a study, the more complicated it is to reveal the real factors contributing to changes in giant panda habitat suitability. Therefore, further understanding of the impacts of climate change on giant pandas is restricted (Pan et al. 2015).

The Minshan Mountains is a focal point for giant panda conservation and provides habitat for 42.8% (797 individuals) of the total giant panda population in China. Climate change is predicted to lead to dry‐warm climates in the future (Wang et al. 2010a), suggesting that the Minshan Mountains region will inevitably be influenced. However, knowledge of climate change‐induced dynamics of distribution patterns and habitat suitability in this region is lacking. This leaves a significant knowledge gap for conservation network efforts. Because fringe areas of ecological patches are sensitive to environmental changes (i.e., the edge effect of ecology) (Russell et al. 2012), we focused on the north Minshan mountains in our assessment.

The goals of this study were to (1) identify the preferred temperature range of giant pandas and their current distribution with respect to climatic regions in the north Minshan mountains; (2) predict variations in the spatial patterns of giant panda habitat distribution and habitat suitability due to climate change in the north Minshan mountains. We also expect our findings to provide useful information for the sustainable protection of giant pandas and their habitat under future climate change.

## Methods

### Study area

**Figure 1:**
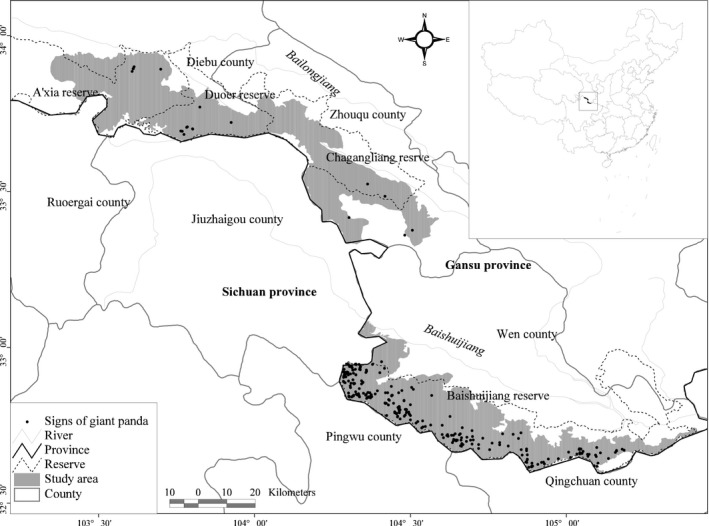
Location of the study area in the north Minshan Mountains. The study area is divided into eastern and northwestern regions.

The study area spans the northernmost distribution area of giant pandas in the Minshan Mountains (103°06′55″ E to 105°36′12″ E and 32°35′44″ N to 34°00′36″ N) (Fig. 1). This region encompasses three and four counties in Gansu Province (Wen County, Diebu County, and Zhouqu County) and in Sichuan Province (Ruoergai, Jiuzhaigou, Pingwu, and Qingchuan) respectively. In this area, there are four giant panda nature reserves: Baishuijiang, Duoer, A'xia, and Chagangliang. The study area is divided into eastern and northwestern regions. The eastern region is located at the south coast of the Baishuijiang River, belonging to the Baishuijiang Nature Reserve, and its elevation ranges from 800 m to 4000 m (average: 2032.7 ± 698.9 m). The northwestern region is located at the southeastern coast of the middle and upper reaches of the Bailongjiang River and contains the Chagangliang, Duoer, and A'xia Nature Reserves. The elevation of the northwest region is 1600 m to 4400 m (average: 3154.3 ± 606.5 m). According to the fourth national giant panda survey, there were 131 giant pandas (16.4% of the total giant panda population in the Minshan Mountains) and 318,169.1 hm^2^ of giant panda habitat in the study area in 2012 (State Forestry Administration, unpublished, 2014).

### Data preparation

The Chinese State and Forestry Administration conducted the fourth national giant panda survey from June to September 2012. Evidence of the presence of giant pandas such as feces, dens, sleeping sites, and footprints were recorded at a frequency of 1 transect per 100 hm^2^. Latitude, longitude, elevation, vegetation types, and bamboo information were recorded for each “sign point”. The fourth national survey collected 278 giant panda signs in our study area. Vegetation and bamboo in the study area were divided into 14 categories and nine species, respectively (Table 1), and elevation and GIS layers were generated using the DEM (digital elevation mode) based on contour lines (1:50,000).

**Table 1:** Suitability of temperature, vegetation, and elevation to giant pandas in the study area

Temperature interval (°C)	Number of signs	Proportion (%)	Suitability	Formation group	Number of signs	Proportion (%)	Suitability	Elevation interval (m)	Number of signs	Proportion (%)	Suitability
−6	2	0.7%	1	Evergreen shrub with leathery leaves	1	0.4%	1	1300	0	0.0%	1
−3	1	0.4%	1	Birch forest	12	4.3%	1	1400	1	0.4%	1
0	2	0.7%	1	Spruce‐fir forest	157	56.5%	3	1500	0	0.0%	1
3	4	1.4%	1	Oak forest	67	24.1%	2	1600	1	0.4%	1
6	12	4.3%	2	Deciduous broad‐leaved forest	24	8.6%	2	1700	2	0.7%	1
9	20	7.2%	2	Montane deciduous broad‐leaved mesic shrub	7	2.5%	1	1800	11	4.0%	2
12	14	5.0%	2	Temperate pine forest	6	2.2%	1	1900	9	3.2%	2
15	27	9.7%	3	Temperate bamboo grove	1	0.4%	1	2000	10	3.6%	2
18	37	13.3%	3	Juniper forest	3	1.1%	1	2100	18	6.5%	2
21	42	15.1%	3	Poplar forest	0	0.0%	1	2200	18	6.5%	2
24	26	9.4%	3	Broad‐leaved mixed forest of hemlock	0	0.0%	1	2300	10	3.6%	2
27	19	6.8%	2	Alpine deciduous broad‐leaved shrub	0	0.0%	1	2400	9	3.2%	2
30	11	4.0%	2	Montane man‐made forest	0	0.0%	1	2500	17	6.1%	3
33	14	5.0%	2	Deciduous broad‐leaved shrub on limestone mountain	0	0.0%	1	2600	25	9.0%	3
36	17	6.1%	2					2700	35	12.6%	3
39	14	5.0%	2					2800	37	13.3%	3
42	10	3.6%	2					2900	29	10.4%	3
45	3	1.1%	1					3000	17	6.1%	3
48	2	0.7%	1					3100	18	6.5%	3
51	0	0.0%	1					3200	7	2.5%	2
54	1	0.4%	1					3300	3	1.1%	1
								3400	0	0.0%	1
								3500	0	0.0%	1
								3600	1	0.4%	1

Temperature has a larger impact on giant pandas than rainfall, and the most influential temperature factor is the mean temperature of the warmest quarter of the year (Songer et al. 2012). We obtained the current mean temperature of the warmest quarter and future temperature projections of the year 2050 from the WorldClim database at resolution of 30 sec (WorldClim.com). A2 was selected as the present CO_2_ emission pattern of greenhouse gas scenarios, indicating a focus on economic development, and hence a high level of CO_2_ emissions. For the year 2050, the RCP (representative concentration pathway) 2.6 was selected as the CO_2_ emission pattern and BCC‐CSM1‐1 as the GCM (global climate model), indicating that CO_2_ emissions will be lowered due to the future development of an environmentally friendly society (Hijmans et al. 2005; Field et al. 2007).

All data were based on the UTM WGS 84 coordinate system, and the raster data resolution was 30 × 30 m.

### Analysis of giant panda temperature preference

Given that interactions between different factors may influence the determination of a specific factor, we chose the mean temperature of the warmest quarter as the temperature proxy with the greatest impact on giant pandas (variable contribution = 36.4%) by referring to Songer et al. (2012). First, the study area was divided into 2 km^2^ grids based on the panda home range diameter (1400 m) (Hu 2001), and the relationship between the number of occurrences and mean temperature of the warmest quarter within each grid was analyzed using Pearson correlation analysis (Fan et al. 2014). Then, we collected temperature data at the sign points based on the WorldClim dataset, and analyzed the sign frequency over temperature intervals of 3°C to determine the temperature preference of giant pandas. We divided the mean temperature range selected by giant pandas (about 60 degrees) into 20 equal intervals (3°C) to match the classification of other habitat factors such as elevation and vegetation for more accurate results. More signs corresponding to a certain temperature interval suggested that the interval was preferred. According to the degree of preference, we divided the suitability of different temperature intervals into three categories: suitable, subsuitable, and ordinary (Table 1).

### Habitat suitability model choice

In order to identify the current habitat suitability and project the future state under climate change in 2050, the habitat suitability model developed by (Store and Kangas (2001)) was employed. This model is considered as a typical method to estimate habitat suitability evaluations over large areas. The modeling approach presented here is a more flexible but realistic standardization approach based on expert knowledge, especially when there is uncertainty inherent in the input data (Wood and Dragicevic 2007). The model has also been extensively used for giant panda habitat suitability evaluations in previous studies (Xu et al. 2006; Gong et al. 2010). The detailed modeling procedure followed the steps reported in the literature (Store and Kangas 2001) with minor modifications. Instead of the MCE program, MAXENT (Phillips and Dudík 2008) was used to calculate the relative importance of each habitat factor.

### Habitat suitability index selection and parameter justification

Both biotic and abiotic factors are important for assessing panda habitat suitability (Liu et al. 1999; Xu et al. 2006). Based on habitat factors used in previous studies (Songer et al. 2012), temperature and other important habitat factors including elevation, vegetation, and bamboo were considered in the habitat suitability assessment (Songer et al. 2012). We created selection criteria for the habitat suitability assessment by combining the proportion of occurrences and expert knowledge. Similar to the above method for temperature preference, the vegetation and elevation of the study area were classified as suitable, subsuitable, and ordinary (Table 1). In the Minshan mountains range, giant pandas feed on several bamboo species, among which *Fargesia denudata* is the most important food source (accounting for 80.5%). Therefore, areas where this species occur are considered suitable habitat for pandas, whereas areas with other bamboo species are considered subsuitable, and areas without bamboo are considered ordinary habitat.

The habitat factors were weighted by calculating the influence of each factor on the model's performance (Table 2). Furthermore, the variables here were already weighted by assigning suitability scores based on the weighting value. The value of the map suitability calculated in this way ranged from 1 (ordinary) to 3 (suitable) (Table 2). We then derived three threshold ranges (suitable, subsuitable, and ordinary) based on the multiplication of three suitability values and weight for each category. The suitability thresholds in the concept framework model were set as ordinary (0–0.019) (e.g., 0.987*0.502*0.421*0.090 = 0.019), subsuitable (0.019–0.042), and suitable (0.042–0.095) (Table 2). Thus, a pixel with a value greater than 0.042 would be classified as highly suitable in the final map. The habitat was also divided into three levels, including ordinary, subsuitable habitat, and suitable habitat based on the suitability thresholds, using the “reclass” function in ArcGIS.

**Table 2:** The habitat variables and their weight, suitable value and threshold value of suitability

Habitat factors	Weight	The category and suitable value of each habitat factor		
		Suitable:3	Subsuitable:2	Ordinary:1
Elevation	0.493	1.480	0.987	0.493
Temperature	0.251	0.754	0.502	0.251
Bamboo	0.211	0.632	0.421	0.211
Vegetation	0.045	0.135	0.090	0.045
Multiplied		0.095	0.019	0.001
Threshold value		0.042–0.095	0.019–0.042^a^	0–0.019

a 0.042 is the results of any two suitable values and two subsuitable value with its weight mutiplied based expert knowledge.

### Assessment of habitat suitability

Following the above habitat suitability procedure, we categorized the current giant panda habitat in our study area as suitable, subsuitable and ordinary using the spatial analysis function of ArcGIS 10.0. Keeping the assessment criteria unchanged, the suitability of giant panda habitats in 2050 was projected based on the predicted temperature. We compared the variations in habitat suitability due to temperature change from 2012 to 2050 in our study area and determined the effects of temperature on giant panda habitats.

To understand the relationship between temperature, elevation, and vegetation, as well as their corresponding variations, we tested the predicted data for every sign point at present and in 2050 using a paired sample *t* test. We also tested whether temperature had an influence on giant panda elevation preference using the Pearson correlation analysis. All the statistical tests were conducted in SPSS 19.0 (IBM SPSS, New York, USA).

### Model validation

Based on the correlation between giant panda presence (from the State and Forestry Administration's fourth national survey in 2012) and habitat type, the accuracy of the habitat suitability assessment was tested using the proportion of signs in suitable and subsuitable habitats (i.e., higher proportion of signs corresponded to higher accuracy). Furthermore, the correlation between the number of panda presence signs and the mean habitat suitability value within each 2‐km^2^ grid was used to test the validation of habitat assessment model.

For further verification of the accuracy of the above habitat suitability results, we also used MAXENT to relate the current giant panda occurrences to the above environment variables and projected future giant panda habitat changes. By comparing the size and percent of each suitability level within the total area with results from a different assessment model, we can make a convincing validation of our study. Due to the occurrence bias across sites, we thinned the set of occurrence records with the delete identical function of ArcGIS to reduce the possibility of the model overestimating environmental conditions at sites with high sampling density and underestimating environmental conditions in areas with low sampling density. After bias removal, the dataset consisted of 131 locations. To further characterize the model performance, we calculated the average test values of the AUC (Area Under the Curve) with different random subsamples (70% training and 30% test data). The AUC value is widely used as an indicator of a model's ability to discriminate between suitable and unsuitable habitat (Warren and Seifert 2011), and the models were considered reliable when AUC > 0.75 (Rebelo and Jones 2010).

## Results

### Changes in Giant Panda temperature preference

**Figure 2:**
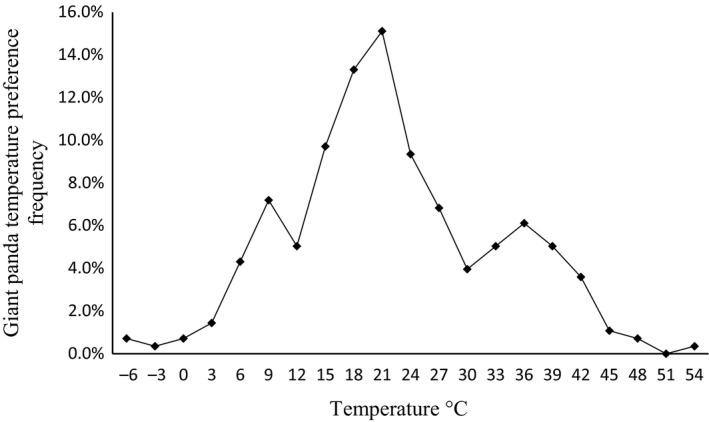
Giant panda temperature preference within the north Minshan Mountains in 2012.

The lowest and highest temperature of the warmest quarter at the sign points was −8.4°C and 53.1°C with a mean temperature of 20.7 ± 10.9°C (Fig. 2). According to the number of signs corresponding to different intervals, the temperature interval preferred by giant pandas was 18–21°C, followed by 15–17°C and 22–24°C. Giant pandas seldom selected other temperature intervals. Therefore, based on expert knowledge and percentage of panda signs, the suitable temperature interval was 15–24°C, the subsuitable intervals were 6–14°C and 25–39°C, and all other temperatures were unsuitable (Table 1). In addition, there was a significant negative correlation between panda signs and temperature in a grid (*ρ *= −0.133, *P* < 0.05), further revealing that giant pandas prefer habitats with relatively low temperatures. Based on the forecast by WorldClim, the mean temperature of the warmest quarter of areas selected by giant pandas will increase up to 28.9 ± 11.0°C by 2050, showing a significant increase (20.7°C vs. 28.9°C, *P* < 0.01) from the present giant panda distribution range.

### Relative importance of habitat factors and current temperature‐suitable giant panda habitats

**Figure 3:**
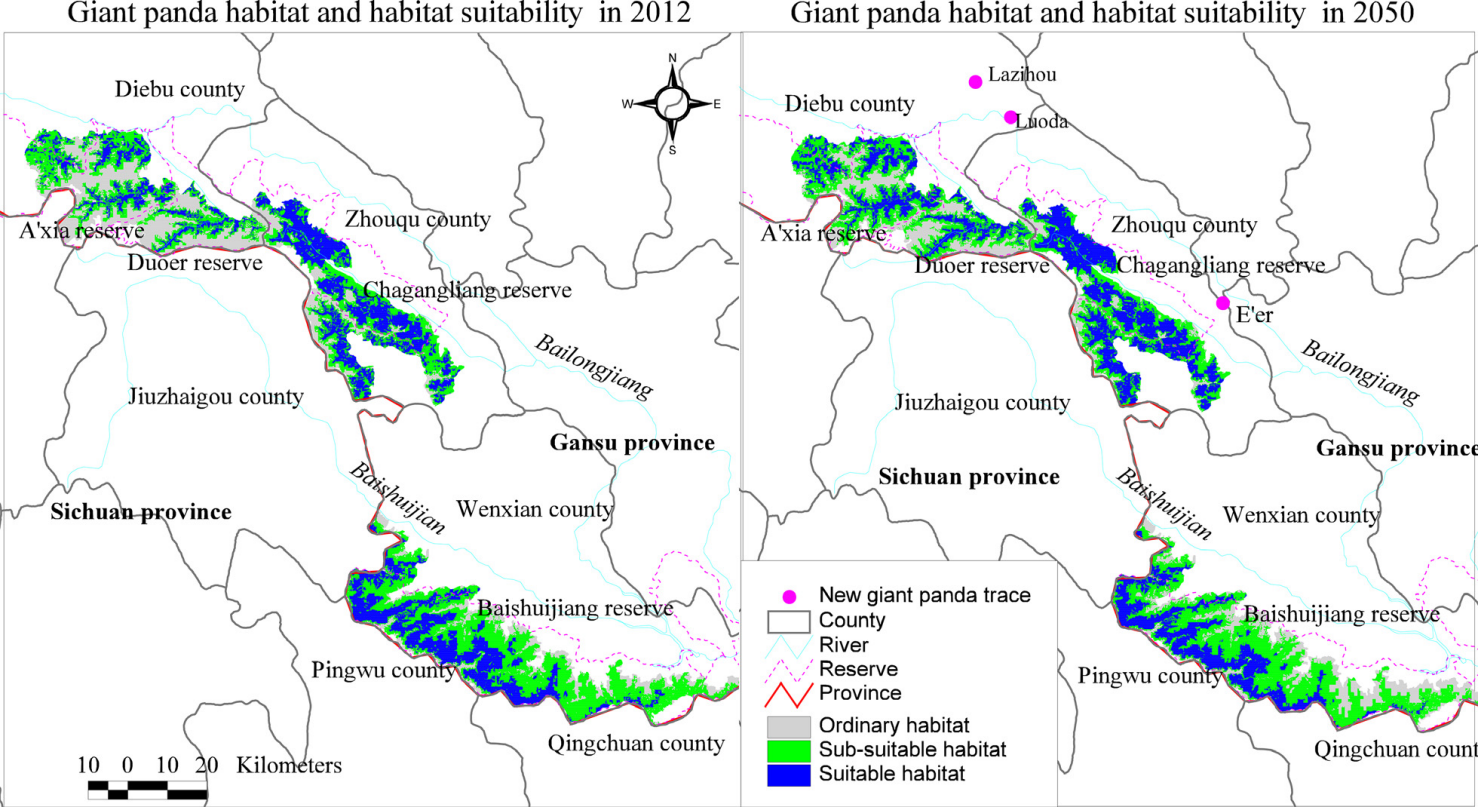
Comparison of the suitability and variations in giant panda habitats in the study area (2012 vs. 2050).

According to the permutation importance from the results of MAXENT model, elevation was the most important predictor of giant panda habitat (49.3%), followed by temperature (25.1%) (Table 2). The results showed that the proportion of the current suitable and subsuitable habitats in our study area was 70.7%, and the habitat suitability was higher in the eastern region than that in the northwest (76.7% vs. 66.3%) (Table 3, Fig. 3).

**Table 3:** Variations in habitat suitability of the east and northwest regions of the study area due to temperature change by the conceptual framework model. The east region includes Baishuijiang reserve, and the northwest region includes three reserves, A'xia, Duoer, and Chagangliang. “%” indicates the proportion of each type of habitat

Habitat type	East region				Northwest region			
	2012		2050		2012		2050	
	Area (hm^2^)	%	Area (hm^2^)	%	Area (hm^2^)	%	Area (hm^2^)	%
Suitable	39,249.5	29.2	35,082.6	26.1	53,655.9	29.2	65,599.9	35.7
Subsuitable	63,847.6	47.5	58,471.0	43.5	68,172.4	37.1	74,236.2	40.4
Ordinary	31,319.0	23.3	40,862.5	30.4	61,924.8	33.7	43,917.0	23.9

### Projection of temperature impacts on habitat suitability in 2050

The temperature in the study area is predicted to increase in 2050 under the IPCC (Intergovernmental Panel on Climate Change) scenario A2, and the suitability of giant panda habitats will increase by 2.7% (Table 3, Fig. 3). Interestingly, the suitability of giant panda habitats in the east and northwest will show opposite variation patterns by 2050. The area of suitable and subsuitable habitats in the Baishuijiang Reserve in the eastern part of the study area will decrease by 9543.5 hm^2^ (6% of the entire reserve area), and the suitability of habitats will decrease by 7.1%, especially in the eastern edge. However, the suitability of habitats in the northwest will increase by 9.8%, and the area of suitable and subsuitable habitats will increase by 18,007.8 hm^2^.

### Model validation

Our results indicated that 274 (98.5%) of the 278 signs found in study area were located in areas of suitable (66.5%) or subsuitable (32%) habitat. Additionally, the correlation between panda presence and habitat suitability was positive (*ρ *= 0.175, *P* < 0.05).Therefore, the methods, factor selection, and standards for the habitat suitability assessment and the habitat assessment model were accurate.

The comparative results between the model and the MAXENT model showed the same change in the direction of habitat suitability impacted by temperature (Tables 3, 4). The MAXENT model also predicted higher habitat suitability in the eastern region than in the northwest at present (56.1% vs. 43.4%). The suitability of giant panda habitats will slightly increase by 2%, and the area of suitable and subsuitable habitats in the eastern part of the study area will decrease by 5.01%. However, the two types of habitat in the northwest will increase by 7.5%. Both the training AUC (0.89) and test AUC (0.79) showed that the performance of the model was reliable. The similar results of the two models suggested that the predicted habitat suitability and its dynamics are highly accurate. This also shows that our methods captured the relationship between giant panda occurrence and spatial distribution of habitat under temperature change.

**Table 4:** Variations in habitat suitability of the east and northwest regions of the study area due to temperature change by Maxent model. The east region includes Baishuijiang reserve, and the northwest region includes three reserves, A'xia, Duoer, and Chagangliang. “%” indicates the proportion of each type of habitat

Habitat type	East region (Baishuijiang reserve)				Northwest region (A'xia, Duoer, and Chagangliang reserves)			
	2012		2050		2012		2050	
	Area (hm^2^)	%	Area (hm^2^)	%	Area (hm^2^)	%	Area (hm^2^)	%
Suitable	35,975.9	26.8	34,178.6	25.4	30,700.1	16.7	33,936.6	18.5
Subsuitable	39,415.0	29.3	34,487.1	25.7	49,004.0	26.7	59,556.6	32.4
Ordinary	59,025.2	43.9	65,750.4	48.9	10,4049.0	56.6	90,259.9	49.1

## Discussion

Temperature requirements for individual growth, development, and survival are thought to be the principal factors limiting the geographic range of giant pandas (Wei et al. 1995; Xu et al. 2006; Wang et al. 2010b). Thus, giant pandas may migrate to more temperature‐suitable habitats in response to temperature change. Our study showed that temperature (relative importance = 25.1%) was the second most important variable influencing giant panda habitat,which is consistent with the results of Songer et al. (2012). However, temperature data with three main habitat parameters (elevation, vegetation type, and bamboo species) was integrated in our study, making the model more synthetic. We found there was a significant negative correlation between temperature and panda presence (*ρ *= −0.133, *P* < 0.05) and temperature preferrence and elevation selection was significantly related (*ρ *= 0.996, *P* < 0.01), which indicates giant pandas will explore higher altitudes as new habitats as the climate becomes drier and warmer. Latest news showed that giant pandas appeared in the townships of Luoda and Lazikou, which are 20 km north of the current distribution area. In 2015, new signs of panda presence appeared in E'er village, which is 8 km from the most suitable habitat areas within the northwest of the study area (news.xinhuanet.com) (Fig. 3). They even found and rescued injured giant pandas in Zhuoni County of the Yellow River Basin, which is further north (Gansu Forestry Department, unpublished, 2014). These facts further suggest that giant pandas in the study area might move toward the northwest (Fan et al. 2014), and also confirm that giant pandas have a strong evolutionary potential to adapt to environmental changes (Zhang et al. 2008; ;Wei et al. 2015). We inferred that more giant panda habitats will exist in high‐altitude and high‐latitude regions owing to increased temperature.

Due to climate change, the overall suitability of giant panda habitats in north Minshan Mountains will increase by 2.7%. However, the quality of the habitat of giant pandas in the existing and potential habitats of the giant pandas, excepting the Qinling mountains, gradually turns worse with climate change (a decrease of 3.16%) (Jian et al. 2014). It can be deduced that the impacts of climate change on giant pandas in north Minshan mountains is not too bad as expected for the following two reasons. First, food is an important driving factor of habitat selection (Nie et al. 2015). For example, for the giant pandas in the Qinling Mountains, climate change might cause a bamboo shortage by the end of the 21st century, which will induce the habitat loss (Tuanmu et al. 2013). In contrast with the Qinling Mountains, there are 400,000 hm^2^ of bamboo groves on the north coast of the Bailongjiang River in the study area (personal communication), and these resources can likely provide enough food for giant panda survival at present and in the future. Secondly, the unsuitable habitat in the northwest Minshan Mountains will turn into suitable habitat because the bamboo distribution and species diversity may increase under climate change (Li et al. 2014). We also discovered the suitability of giant panda habitats in the eastern and northwestern regions of the study area showed opposite variation patterns due to climate change (Tables 3, 4). More giant panda habitats will appear due to increasing temperature (Fig. 3). However, under dry and warm climate stress, giant pandas compelled to disperse to new habitats may be influenced by many natural and artificial factors such as geographical barriers, ecological corridors, human disturbances, and reserve planning. These influences require further in‐depth study.

The comparative results between the model used and the MAXENT model showed the similar change in the direction of habitat suitability impacted by temperature (Tables 3, 4). A slight variation existed, such as the overall suitability (2.7% increase for the model used and 2% increase for the MAXENT model), because the range of assessment criteria we selected was relatively extensive. On the other hand, comprehensive dataset and tuned parameters prevent MAXENT from matching the input data too closely, which is known as “overfitting” and has a detrimental effect on predictive performance (Hastie et al. 2005). The validation results indicated the model used is a simple and convenient tool to identify the impacts of climate change on wildlife habitat, especially where there is uncertainty inherent in the input data (Wood and Dragicevic 2007). With the continued implementation of giant panda conservation and natural forest protection by the Chinese government (Li et al. 2013), climatic variables may become the most important factors influencing giant panda habitat compared with infrequent natural disasters such as earthquakes and mudslides. Hence, the calculation and forecasting of climatic factors will directly determine the accuracy of giant panda research and the effectiveness of protective measures. However, current climate‐monitoring stations in China are generally far from giant panda habitats, and therefore high quality climatic data are difficult to obtain. Thus, the construction of climate monitoring and collection systems in giant panda habitats should be enhanced in future conservation projects to provide accurate raw data to meet the demands of protection and research agencies. Such measures will allow these groups to effectively cope with the effects of global climate change on giant pandas.

Our findings strongly imply that giant panda will disperse to northwestern Minshan Mountains under climate change. This region may become the important potential area where giant panda populations take refuge. Reintroduction of captive pandas and restoration of small populations should be attempted. Such measures will strengthen current populations, as well as benefit giant panda population dispersal and development in the future. However, the Baishuijiang Reserve, the largest giant panda reserve and population distribution area (population size: 110) located in the north Minshan Mountains, faces the severe threat of habitat reduction, and giant panda populations may move to the west region of this reserve. The administration of this reserve should take preemptive measures by launching habitat recovery programs in the east of the reserve to improve the quality and integrity of habitats, and by constructing migration corridors to allow giant pandas to disperse to new suitable habitats. Moreover, population monitoring should be strengthened in the west of the reserve, along with assessments of environmental capacity and population risk. The cooperation between reserves and nearby regions should be improved to facilitate giant panda dispersal and communication in and around the Baishuijiang Reserve, thereby guaranteeing population safety.

## Conflict of Interest

None declared.
